# Solving the chemical master equation using sliding windows

**DOI:** 10.1186/1752-0509-4-42

**Published:** 2010-04-08

**Authors:** Verena Wolf, Rushil Goel, Maria Mateescu, Thomas A Henzinger

**Affiliations:** 1 Computer Science Department, Saarland University, Saarbrücken, Germany; 2 Department of Computer Science and Engineering, IIT Bombay, Bombay, India; 3 School of Computer and Communication Sciences, EPFL, Lausanne, Switzerland; 4 Institute of Science and Technology, Klosterneuburg, Austria

## Abstract

**Background:**

The chemical master equation (CME) is a system of ordinary differential equations that describes the evolution of a network of chemical reactions as a stochastic process. Its solution yields the probability density vector of the system at each point in time. Solving the CME numerically is in many cases computationally expensive or even infeasible as the number of reachable states can be very large or infinite. We introduce the sliding window method, which computes an approximate solution of the CME by performing a sequence of local analysis steps. In each step, only a manageable subset of states is considered, representing a "window" into the state space. In subsequent steps, the window follows the direction in which the probability mass moves, until the time period of interest has elapsed. We construct the window based on a deterministic approximation of the future behavior of the system by estimating upper and lower bounds on the populations of the chemical species.

**Results:**

In order to show the effectiveness of our approach, we apply it to several examples previously described in the literature. The experimental results show that the proposed method speeds up the analysis considerably, compared to a global analysis, while still providing high accuracy.

**Conclusions:**

The sliding window method is a novel approach to address the performance problems of numerical algorithms for the solution of the chemical master equation. The method efficiently approximates the probability distributions at the time points of interest for a variety of chemically reacting systems, including systems for which no upper bound on the population sizes of the chemical species is known a priori.

## Background

Experimental studies have reported the presence of stochastic mechanisms in cellular processes [1-9] and therefore, during the last decade, *stochasticity *has received much attention in systems biology [10-15]. The investigation of stochastic properties requires that computational models take into consideration the inherent randomness of chemical reactions. Stochastic kinetic approaches may give rise to dynamics that differ significantly from those predicted by deterministic models, because a system might follow very different scenarios with non-zero likelihoods.

Under the assumption that the system is spatially homogeneous and has fixed volume and temperature, at a each point in time the state of a network of biochemical reactions is given by the population vector of the involved chemical species. The temporal evolution of the system can be described by a Markov process [16], which is usually represented as a system of ordinary differential equations (ODEs), called the *chemical master equation *(CME).

The CME can be analyzed by applying numerical solution algorithms or, indirectly, by generating trajectories of the underlying Markov process, which is the basis of Gillespie's *stochastic simulation algorithm *[17,18]. In the former case, the methods are usually based on a matrix description of the Markov process and thus primarily limited by the size of the system. A survey and comparisons of the most established methods for the numerical analysis of discrete-state Markov processes are given by Stewart [19]. These methods compute the probability density vector of the Markov process at a number of time points up to an a priori specified accuracy. If numerical solution algorithms can be applied, almost always they require considerably less computation time than stochastic simulation, which only gives estimations of the measures of interest. This is particularly the case if not only means and variances of the state variables are estimated with stochastic simulation, but also the probability of certain events. However, for many realistic systems, the number of reachable states is huge or even infinite and, in this case, numerical solution algorithms may not be applicable. This depends mainly on the number of chemical species. In low dimensions (say <10) a direct solution of the CME is possible whereas in high dimensions stochastic simulation is the only choice. In the case of stochastic simulation estimates of the measures of interest can be derived once the number of trajectories is large enough to achieve the desired statistical accuracy. However, the main drawback of simulative solution techniques is that a large number of trajectories is necessary to obtain reliable results. For instance, in order to halve the confidence interval of an estimate, four times more trajectories have to be generated. Consequently, often stochastic simulation is only feasible with a very low level of confidence in the accuracy of the results.

**Figure 1:**
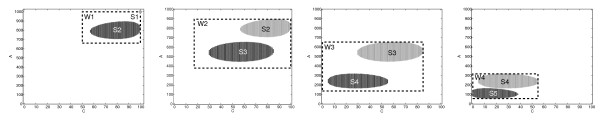
**The sliding window method**. In each iteration step, the window *W*_*i *_captures the set *S*_*i *_of states in which the significant part of the probability mass is initially located (light gray), the set *S*_*i*+1 _of states that are reached after a time step (dark gray), as well as the states that are visited in between.

In this paper, we mitigate the performance problems of numerical solution algorithms for the CME. Instead of a global analysis of the state space, we propose the *sliding window method*, which comprises a sequence of analyzes local to the significant parts of the state space. In each step of the sequence, we dynamically choose a time interval and calculate an approximate numerical solution for a manageable subset of the reachable states. In order to identify those states that are relevant during a certain time period, for each chemical species, we estimate an upper and lower bound on the population size. This yields the boundaries of a "window" in which most of the probability mass remains during the time interval of interest. As illustrated in Figure 1, the window "slides" through the state space when the system is analyzed in a stepwise fashion. In each step, the initial conditions are given by a vector of probabilities (whose support is illustrated in light gray), and a matrix is constructed to describe the part of the Markov process where the window (illustrated by the dashed rectangular) is currently located. Then the corresponding ODE is solved using a standard numerical algorithm, and the next vector (illustrated in dark gray) is obtained.

We focus on two specific numerical solution methods, the *uniformization method *and the *Krylov subspace method*. We compare their efficiency when they are used to solve the ODEs that arise during the sliding window iteration. We also compare the sliding window method to the numerical algorithms applied in a global fashion, that is, to all reachable states (not only to the states of the window), for systems of tractable size. We are interested in the probability distribution of the Markov process and not only in means and variances. These probabilities are difficult to estimate accurately with stochastic simulation. Therefore, we compare the solution obtained by the sliding window method only to numerical solution algorithms but not to stochastic simulation.

Recently, *finite state projection algorithms *(FSP algorithms) for the solution of the CME have been proposed [20,21]. They differ from our approach in that they are based solely on the structure of the underlying graph, whereas the sliding window method is based on the stochastic properties of the Markov process. The FSP algorithms start with an initial projection, which is expanded in size if necessary. The direction and the size of the expansion is chosen based on a qualitative analysis of the system in a breadth-first search manner. It is not clear how far the state space has to be explored in order to capture most of the probability mass during the next time step. Thus, if the projection size is too small, the computation has to be repeated with an expanded projection. Moreover, for most models, the location of the main portion of the probability mass follows a certain direction in the state space, whereas the expansion is done in all directions. Therefore, unnecessary calculations are carried out, because the projection contains states that are visited with a small probability. By contrast, in the sliding window approach, we determine the location and direction of the probability mass for the next computation step based on the reaction propensities and the length of the time step. The projection that we obtain is significantly smaller than the projection used in the FSP whereas the accuracy of our approach is similar to the accuracy of the FSP. In this way we achieve large memory and computational savings, since the time complexity of our window construction is small compared to the calculation of the probability distribution of the window. In our simulations we never had to repeat the computation of the probabilities using a window of larger size.

The Fokker-Planck equation is an approximation of the CME, for which a solution can be obtained efficiently [22,23]. This approximation, however, does not take into account the discrete nature of the system, but changes the underlying model by assuming a continuous state space. Other approaches to approximate the probability distributions defined by the CME are based on sparse grid methods [24], spectral methods [25], or the separation of time scales [26,27]. The latter approach uses a quasi-steady state assumption for a subset of chemical species and calculates the solution of an abstract model of the system. In contrast, we present an algorithm that computes a direct solution of the CME. Our method is also related to tau-leaping techniques [18,28], because they require estimates of the upper and lower bounds on the population sizes of the chemical species, just as our method. The *time leap *must be sufficiently small such that the changes in the population vector do not significantly affect the dynamics of the system. Our method differs from the calculation of the leap in predicting the future dynamics for a dynamically chosen time period. More precisely, we determine the length of the next time step while approximating the future behavior of the process.

Here, we present the sliding window method in more detail and provide an additional comparison between uniformization and Krylov subspace methods for the solution of the window. Moreover, we have improved our implementation of the algorithm and evaluated it on more examples, such as the bistable toggle switch, which is reported in detail.

The remainder of this paper is organized as follows. We first describe the theoretical framework of our modeling approach in the Background Section. In the Results Section we present the sliding window method, and numerical solution approaches for the CME. Experimental results are given at the end of Section Results.

### Stochastic model

We model a network of biochemical reactions as a Markov process that is derived from the stochastic chemical reaction kinetics [16,29]. A physical justification of Markovian models for coupled chemical reactions has been provided by Gillespie [17]. We consider a fixed reaction volume with *n *different chemical species that is spatially homogeneous and in thermal equilibrium. A state of the system is given by a vector *x *∈ , where the *i*-th entry, denoted by *x*_*i *_describes the number of molecules of type *i*. We assume that molecules collide randomly and chemical reactions occur at random times. By *R*_1_, ..., *R*_*k*_, we denote the different types of chemical reactions and with each type *R*_*m*_, *m *∈ {1, ..., *k*}, we associate a *propensity function α*_*m *_:  → ℝ_≥0_. The propensity function is of the form(1)

where *c*_*m *_> 0 is a constant and *l*_*i *_is the number of molecules of type *i *that are consumed by a reaction of type *R*_*m*_. The propensity *α*_*m*_(*x*) determines the "speed" of the reaction *R*_*m *_in *x*, as explained below. Note that  equals the number of all distinct combinations of reactants. Besides the propensity function, we associate a *change vector v*^(*m*) ^∈ ℤ^*n *^with *R*_*m *_that describes the effect of reaction type *R*_*m*_. If *x *is the current state and *α*_*m*_(*x*) > 0 then *x *+ *v*^(*m*) ^is the state of the system after a reaction of type *R*_*m*_. Note that *α*_*m*_(*x*) > 0 implies that *x *+ *v*^(*m*) ^contains no negative entries.

We denote the initial state of the system by *y *∈  and define *S *⊆  as the set of all states reachable from *y *via an arbitrary number of reactions, that is, *S *is the smallest subset of  such that *y *∈ *S *and *x' *∈ *S *iff there exists *m *∈ {1, ..., *k*} and *x *∈ *S *with *α*_*m*_(*x*) > 0 and *x *+ *v*^(*m*) ^= *x'*. Note that *S *is countable but possibly infinite.

**Example 1 ***We describe an *enzyme-catalyzed substrate conversion *by the three reactions R*_1 _: *E *+ *S *→ *ES*, *R*_2 _: *ES *→ *E *+ *S*, *R*_3 _: *ES *→ *E *+ *P. This network involves four chemical species, namely, enzyme (E), substrate (S), complex (ES), and product (P) molecules. The change vectors are v*^(1) ^= (-1, -1, 1, 0), *v*^(2) ^= (1, 1, -1, 0), *and v*^(3) ^= (1, 0, -1, 1)*. For *(*x*_1_, *x*_2_, *x*_3_, *x*_4_) ∈ , *the propensity functions are*

*As above, the set of states reachable from the initial state y *= (*y*_1_, *y*_2_, *y*_3_, *y*_4_) *is finite because of the conservation laws y*_1 _= *x*_1 _+ *x*_3 _*and y*_2 _= *x*_2 _+ *x*_3 _+ *x*_4_*, where we assume that y*_3 _= *y*_4 _= 0.

**Example 2 ***We consider a gene expression model *[12], *which involves two chemical species, namely, mRNA (M) and protein (P). Transcription of a gene into mRNA is modeled by reaction R*_1 _: ∅ → *M, translation of mRNA into protein by R*_2 _: *M *→ *M *+ *P, degradation of mRNA by R*_3 _: *M *→ ∅*, and degradation of proteins by R*_4 _: *P *→ ∅. *A state is a pair *(*x*_*M*_, *x*_*P*_) ∈ . *If we assume that initially there are no mRNA molecules and no proteins in the system, i.e., y *= (0, 0), *then S *= *and thus infinite. The propensity functions are*

### Chemical master equation

We define a time-homogeneous, regular Markov process [30] (CTMC) (*X*(*t*), *t *∈ ℝ_≥0_) with state space *S *⊆ . We assume that the state changes of *X *are triggered by the chemical reactions. Let *y *be the initial state of *X*, which means that *Pr*(*X*(0) = *y*) = 1. We assume that the probability of a reaction *R*_*m *_occurring in the next infinitesimal time interval [*t*, *t *+ *τ*), *τ *> 0 is given by

For *x *∈ *S *we define the probability that *X *is in state *x *at time *t *by *p*^(*t*)^(*x*) = *Pr*(*X*(*t*) = *x *| *X*(0) = *y*). The *chemical master equation *(CME) describes the behavior of *X *by the differential equation [29](2)

In the sequel, a matrix description of Eq. (2) is more advantageous. It is obtained by defining the *infinitesimal generator matrix Q *= (*Q*(*x*, *x'*))_*x*, *x'*∈*S *_of the CTMC *X *by

where we assume a fixed enumeration of the state space. Note that the row sums of the (possibly infinite) matrix *Q *are zero and *λ*_*x *_= -*Q*(*x*, *x*), the *exit rate *of state *x*, is the reciprocal value of the average residence time in *x*.

Let *T*^(0) ^be equal to the identity matrix *I*, and, for *τ *> 0, let *T*^(*τ*) ^be the transition probability matrix for step *τ *with entries *T*^(*τ*)^(*x*, *z*) = *Pr*(*X*(*t *+ *τ*) = *z *| *X*(*t*) = *x*). The elements of *T*^(*τ*) ^are differentiable and *Q *is the derivative of *T*^(*τ*) ^at *τ *= 0. If *Q *is given and *X *is known to be regular, *T*^(*τ*) ^is uniquely determined by the *Kolmogorov backward and forward equations*(3)

with the general solution *T*^(*τ*) ^= *e*^*Qτ*^. Let ***p***^(*t*) ^be the row vector with entries *p*^(*t*)^(*x*) for *x *∈ *S*. Then the vector form of the CME is(4)

If sup_*x*∈*S *_*λ*_*x *_< ∞, Eq. (4) has the general solution(5)

where the matrix exponential is given by *e*^*Qt *^= .

In the sequel, we will exploit the fact that the set {*T*^(*τ*)^|*τ *≥ 0} is a *transition semi-group *and satisfies the *Chapman-Kolmogorov equations *[30] for all *τ*_1_, *τ*_2 _≥ 0. Let *t*_0_, ..., *t*_*r *_∈ ℝ_≥0 _be such that *t*_0 _< ⋯ <*t*_*r*_. Then,(6)

This means that, for *t*_0 _= 0 and *t*_*r *_= *t*, we obtain ***p***^(*t*) ^by the iterative scheme in Eq. (3) for *t*_1 _- *t*_0_, *t*_2 _- *t*_1_, ..., *t*_*r *_- *t*_*r*-1_.

If the state space if infinite we can only compute approximations of ***p***^(*t*)^. But even if *Q *is finite, several factors can hamper the efficient solution of the matrix exponential in Eq. (5). First of all, the size of the matrix *Q *might be large because it grows exponentially with the number of state variables. However, usually *Q *is sparse, as the number of reaction types is small compared to the number of states. But even when *Q *is sparse often only an approximate solution can be computed efficiently. Adding up a sufficiently large number of terms of the infinite sum  is numerically unstable, as *Q *contains strictly positive and negative entries, leading to severe round-off errors [31]. Various numerical solution methods exist for systems of first-order linear equations of the form of Eq. (4). However, many of them are not useful as they do not preserve the sparseness of *Q*. Several surveys and comparisons exist in literature [19,32,33]. Most popular are methods based on uniformization [34,35], approximations in the Krylov subspace [36], or numerical integration [37,38]. We will describe the former two methods in more detail in the section on numerical solution methods.

## Results

### Sliding window method

The key idea of the algorithm proposed in this paper is to calculate an approximation  of ***p***^(*t*) ^in an iterative fashion, as described in Eq. (6). More precisely, we compute a sequence of approximations  such that for a subset *W*_*j *_of the state space, *j *∈ {1, ..., *r*},  for all *x *∈ *W*_*j*_. The sets *W*_1_, ..., *W*_*r *_are called *windows*, and we assume that *W*_*j *_contains the states at which (most of) the probability mass is concentrated during the time interval [*t*_*j*-1_, *t*_*j*_). We discuss the construction of the window *W*_*j *_later.

Let *Q*_*j *_be the matrix that refers to *W*_*j*_, i.e., we define *Q*_*j*_(*x*, *x'*) = *Q*(*x*, *x'*) if *x*, *x' *∈ *W*_*j*_, and *Q*_*j*_(*x*, *x'*) = 0 otherwise. Note that for the simplicity of our presentation we keep a fixed enumeration of *S *and assume that each *Q*_*j *_has the same size as *Q*. However, the implementation of the method considers only the finite submatrix of *Q*_*j *_that contains entries of states in *W*_*j*_. For *τ*_*j *_= *t*_*j *_- *t*_*j*-1_, we define(7)

where  = (**1**_*y*_)^*T *^and *D*_*j *_is the diagonal matrix whose main diagonal entries are one for *x *∈ *W*_*j *_and zero otherwise. The row vector (**1**_*y*_)^*T *^is one at position *y *and zero otherwise.

In the *j*-th step, the matrix  contains the probabilities to move in *τ*_*j *_time units within *W*_*j *_from one state to another. As initial probabilities, Eq. (7) uses the approximations  for all states *x *∈ *W*_*j*_. The diagonal matrix ensures that the probability mass located in *W*_*j*-1_\*W*_*j *_is ignored during the computation, that is, only elements of the intersection *W*_*j*-1 _∩ *W*_*j *_can have nonzero entries in the vector *D*_*j*_. This is necessary because *Q*_*j *_does not contain the transition rates of states outside of *W*_*j *_(these states are absorbing). Intuitively, the vector  describes the location of the probability mass after moving within *W*_*j*_.

Although *Q*_*j *_is not the generator of a CTMC, Eq. (7) has a simple interpretation for all states *x *∈ *W*_*j*_. Let us fix *j *for the moment, and let the CTMC  be identical to *X*, except that all states *x' *∉ *W*_*j *_are absorbing (i.e., once *x' *is reached, it cannot be left). Let the initial probability distribution of  be such that  for all *x *∈ *W*_*j*_. Then , for all *x *∈ *W*_*j*_. For all *j*, the vectors  are substochastic, and the sum of their entries decreases in each step, i.e,

Probability mass is "lost", because we do not consider the entries  for *x *∈ *W*_*j*-1_\*W*_*j*_, as we multiply with *D*_*j*_. In addition, we lose the probability to leave *W*_*j *_within the next *τ*_*j *_time units because  is a substochastic matrix. If, for all *j*, during the time interval [*t*_*j*-1_, *t*_*j*_) most of the probability mass remains within *W*_*j*_, then the approximation error  is small for all *x *∈ *S*. The probability mass that is lost after *j *steps due to the approximation is given by(8)

Thus, if Eq. (7) is solved exactly, the total approximation error of the sliding window method is *η*_*r*_. Note that the error in Eq. (8) is the sum of the errors of all components of the vector .

#### Window construction

In each step of the iteration the window *W*_*j *_must be chosen such that the error *η*_*j *_is kept small. This is the case if *W*_*j *_satisfies the following conditions: (a) with a sufficiently high probability *X*(*t*_*j*-1_) ∈ *W*_*j*_, (b) the probability of leaving *W*_*j *_within the time interval [*t*_*j*-1_, *t*_*j*_) is sufficiently small.

Requirement (a) implies that *W*_*j *_contains a significant part of the support of , that is, a subset *S*_*j *_⊂ *S *such that  is small. In the first step we set *S*_1 _= {*y*}. For *j *> 1, the window *W*_*j *_is constructed after  is calculated. We fix a small *δ *> 0 and choose *S*_*j *_= {*x *|  > *δ*}. If the support of  is large and distributes almost uniformly, it may be necessary to construct *S*_*j *_such that  is smaller than some fixed threshold. However, our experimental results show that using a fixed threshold yields good results, which makes the additional effort of sorting the support of  unnecessary in practice. Note that requirement (a) implies that *W*_*j *_and *W*_*j*-1 _intersect. Thus, in each step we "slide" the window in the direction that the probability mass is moving.

The sequel of this section focuses on requirement (b), where it is necessary to predict the future behavior of the process. One possibility to find a set *W*_*j *_that satisfies the requirements is to carry out stochastic simulation for *t*_*j *_- *t*_*j*-1 _time units with initial states in *S*_*j*_. This may be costly if we aim at an accurate approximation. Most simulation runs correspond to the average behavior of the system. However, there may be events that are less frequent, but that still have a significant probability. Therefore, we propose an idea that relies on a state-continuous deterministic approximation that, given an initial state *z *∈ *S*_*j*_, estimates the maximal and minimal values each state variable can take during the next *τ*_*j *_= *t*_*j *_- *t*_*j*-1 _time units. More precisely, for each dimension *d *∈ {1, 2, ..., *n*}, we calculate values ,  ∈ ℤ such that

is small, where *X*_*d*_(*τ*) is the *d*-th component of the random vector *X*(*τ*).

The computation of the extreme values ,  is carried out for several states *z*, which are chosen uniformly at random. Our experimental results indicate that the accuracy of our results does not increase when more than 10 states are considered. Let *A*_*j *_⊆ *S*_*j *_be the set of random states. By computing  and , we obtain estimates for the maximal and minimal values of each state variable during the time interval [*t*_*j*-1_, *t*_*j*_) under the condition that *X*(*t*_*j*-1_) ∈ *S*_*j*_. Window *W*_*j *_is now constructed as the union of *S*_*j *_and all states within  and , that is, *W*_*j *_equals(9)

For a fixed state *z *∈ *A*_*j *_we exploit the regular structure of the Markov chain for the computation of  and . We start in state *z *and update the state variables one by one. We assume that for a small time interval of length Δ the rate of reaction type *R*_*m *_remains constant, i.e., is equal to *α*_*m*_(*z*). Then the number of *R*_*m*_-transitions within the next Δ time units is Poisson distributed with parameter *α*_*m*_(*z*)Δ. We can approximate this number by the expectation *α*_*m*_(*z*)Δ of the Poisson distribution. Note that the above assumption is warranted since in the case of coupled chemical reactions the propensities *α*_*m*_(*x*) are linear or at most quadratic in *x*, if only elementary reactions are considered, i.e. reactions that correspond to a single mechanistic step and have therefore at most two reactants. In general, reactions may have intermediate products and/or parallel reaction pathways. They can, however, always be decomposed into elementary reactions. As we are interested in an upper and a lower bound, we additionally consider the standard deviation  of the Poisson distribution. We assume that, if the current state is *x*, within Δ units of time

• at least ,

• at most 

transitions of type *R*_*m *_are taken. Note that if, for instance, *α*_*m*_(*z*)Δ = 1, then we have a confidence of 91.97 percent that the real number of reactions lies in the interval(10)

Let *κ*_*m *_∈  and *l *= 0, 1,.... The iteration(11)

yields continuous deterministic worst-case approximations of *X*(*t *+ Δ), *X*(*t *+ 2Δ),... under the condition that *X*(*t*) = *z*. For functions *α*_*m *_that grow extremely fast in the state variables, the iteration may yield bad approximations because it is based on the assumption that the propensities are constant during [0, Δ).

In the context of biochemical reaction networks, *α*_*m *_is at most quadratic and therefore the approximation given by Eq. (11) yields adequate results. For a given system, we perform the approximation in Eq. (11) for all possible combinations . It is possible to skip combinations that treat preferentially transition types leading to opposite directions in the state space, because they will not give a worst-case bound. Consider, for instance, Example (1) with *c*_1 _= *c*_2 _= *c*_3 _= 1, *z *= (10, 10, 100, 0), and Δ = 0.01. If we assume that more reactions of type *R*_2 _and *R*_3 _happen (than on average) and fewer of *R*_1_, we get , and . This means that the number of complex molecules decreases and *x*^(1) ^= (14,12, 96, 2). We can omit combinations that contain both  and . As *R*_1 _equates *R*_2 _and vice versa, these combinations will not yield good approximations of the extreme values of the state variables. In general, the dependency graph of the reaction network may be helpful to identify those combinations that maximize a certain population (see also the section on experimental results).

In the sequel, each chosen combination is referred to as a *branch *because, for fixed *z*, the corresponding iterations lead to different successors *x*^(*l*+1)^. Note that for a particular branch, for each *m *∈ {1, ..., *k*} we fix *κ*_*m *_= , or *κ*_*m *_=  for all *l*. The iteration ends after ⌈*τ*_*j*_/Δ⌉ steps (where the length of the last time step is the remainder instead of Δ), and the extreme values  and  are given by the minimal and maximal values of the state variables during the iteration. More specifically,  and , where 1 ≤ *d *≤ *n*, *x*^(*l*) ^= , and *z *= *x*^(0)^.

The calculation of  and  is described in pseudocode in Table 1 (left column), called *ContDetApprox*. Note that the superscript *i *refers to the current branch and not to the iteration in Eq. (11) which is carried out in line 19. The number of branches is 2*n *as maximal and minimal values for each dimension are necessary. In line 17, we decide, depending on the current branch *i*, whether *κ*_*m *_is set to , or .

**Table 1:** The method *ContDetApprox *(left) and the main procedure *sWindow *(right)

ALGORITHM *ContDetApprox *(*z*, *τ*, *b*^**+**^, *b*^**-**^)	ALGORITHM *sWindow*(*y*, *t*, ϵ, *δ*)
Input: initial state *z*, length *τ *of time interval, old boundaries	Input: initial state *y*, times *t *= (*t*_0_, ..., *t*_*r*_), error ϵ > 0, threshold *δ *> 0
Output: new boundaries	Output: probability vectors ***p***_0_, ..., ***p***_*r*_

1 **for **each branch *i *∈ {1, ..., 2*n*} **do**	1 ***p***_0 _= (**1**_*y*_)^*T*^; //*start with probability one in y*
2 *x*^⟨*i*⟩^:= *z*; //*z is initial state of all branches*	2 **for ***j *:= 1 to *r ***do**
3 **end**	3 *τ*_*j *_:= *t*_*j *_- *t*_*j*-1_; //*length of next time step*
4 *time *:= 0;	//*define S*_*j*_*for construction of W*_*j*_
5 Δ := *step_size*; //*choose length of time steps*	4 *S*_*j *_:= {*x *| ***p***_*j*-1_(*x*) >*δ*};
6 **while ***time *<*τ ***do**	5 *numStates *:= min(10, *size*(*S*_*j*_));
7 **for **each branch *i *∈ {1, ..., 2*n*} **do**	//*choose numStates random states from S*_*j*_
//*compare current state variables with boundaries*	6 *A*_*j *_:= *rand*(*S*_*j*_, *numStates*);
8 **for ***d *= 1 to *n ***do**	7 *b*^+^: -∞; *b*^- ^: +∞; //*initial boundaries*
9 **if ****then**	8 **for **each *z *in *A*_*j *_**do**
10 ; //*adjust upper bound*	//*run continuous determ. approximation*
11 **end**	//*on z and update boundaries*
12 **if ****then**	9 (*b*^+^, *b*^-^) := *ContDetApprox *(*z*, *τ*_*j*_, *b*^+^, *b*^-^);
13 ; //*adjust lower bound*	10 **end**
14 **end**	11 *Q*_*j *_:= *generator *(*S*_*j*_, *b*^+^, *b*^-^); //*construct Q*_*j*_
15 **end**	//*construct diagonal matrix for W*_*j *_(*cf*. *Eq*. (7))
16 **for ***m *:= 1 to *k ***do**	12 *D*_*j *_:= *diag*(*S*_*j*_, *b*^+^, *b*^-^);
//*choose more*/*fewer transitions of type R*_*m*_	13 ; //*solve Eq*. (7)
//*depending on branch i*	14 **end**
17 *κ*_*m *_:= *choose*(*α*_*m*_(*x*^⟨*i*⟩^)·Δ, *i*);	
18 **end**	
19 ; //*update state *(*cf*. *Eq*. (11))	
20 **end**	
21 *time *:= *time *+ Δ;	
22 **end**	

Regarding the choice of the time step Δ, we suggest to choose Δ dynamically such that for each *m *the interval in Eq. (10) covers at least, say, 80% of the probability mass of the corresponding Poisson distribution. Clearly, the accuracy of the method increases in the case of larger intervals covering more probability mass. For our experimental results, we chose Δ such that *λ*_*x*_·Δ = 1 yielded sufficiently accurate results.

#### Sliding window algorithm

In the right column of Table 1 we describe the main procedure, called *sWindow*, in pseudocode. The for loop in lines 2-14 implements the approximations of  by successively computing vector ***p***_*j *_from ***p***_*j*-1_. Input ϵ is a bound for the total approximation error caused by the solutions of the ODEs in line 13. The array *t *contains the time instances *t*_0_, ..., *t*_*r*_. For our experimental results, we compare two different time stepping mechanisms that are explained below. The parameter *δ *is the threshold that is used to remove those states in the support of *p*_*j*-1 _having a smaller probability than *δ*. We define *S*_*j *_as the set of all states *x *for which *p*_*j*-1_(*x*) is greater than *δ *in line 4. Note that for *j *= 1 the set *S*_1 _contains only the initial state *y*. In line 6, *rand*(*S*_*j*_, *numStates*) returns a set of *numStates *random elements from *S*_*j *_that are used to construct the vectors *b*^+ ^and *b*^- ^in lines 7-10. The rate entries of all states in the window *W*_*j *_(cf. Eq. (9)) are calculated in line 11, and all remaining entries in *Q*_*j *_are set to zero. A solution method is invoked in line 13 to calculate *p*_*j *_from *p*_*j*-1_. This can be, for instance, the uniformization method, an ODE solver or a method based on an approximation in the Krylov subspace. We pass a time step of length *τ*_*j *_and the corresponding fraction  of the approximation error.

We can calculate the overall loss of probability mass from the output *p*_*r *_by *η*_*r *_= 1 - ∑_*x*_*p*_*r*_(*x*). This value includes both approximation errors of the algorithm: (1) the probability of leaving window *W*_*j *_during the time interval [*t*_*j*-1_, *t*_*j*_) and (2) the probability  that is lost due to the sliding of the window, obtained by the multiplication with *D*_*j *_(cf. Eq. (7)).

Note that it is always possible to repeat a computation step in order to increase the obtained accuracy. More precisely, we can determine a larger window by increasing the confidence of the interval in Eq. (10), i.e. by choosing the time step Δ such that for each *m *the maximal/minimal number of transitions of type *R*_*m *_lies in the interval with a certain confidence (e.g. with a confidence of 80%). For our experimental results, however, we did not repeat any computation step since we always obtained sufficiently accurate results.

#### Time intervals

For our experimental results, we compare two different time stepping mechanisms for Algorithm *sWindow *(see Table 1, right). We either choose equidistant time steps *τ*_*j *_= *τ*, for all *j*, or we determine *τ*_*j *_during the construction of the window *W*_*j *_(adaptive time steps). The latter method yields faster running times. Depending on the dynamics of the system, long time steps may cause three problems: (1) the window is large and the size of the matrix *Q*_*j *_may exceed the working memory capacity, (2) the dynamics of the system may differ considerably during a long time step and *Q*_*j *_has bad mathematical properties, (3) the window may contain states that are only significant during a much shorter time interval. If, on the other hand, the time steps are too small then many iterations of the main loop have to be carried out until the algorithm terminates. The windows overlap nearly completely, and even though each step may require little time, the whole procedure can be computationally expensive. One possibility is to fix the size of the windows and choose the time steps accordingly. But this does not necessarily result in short running times of the algorithm either. The reason is that the time complexity of the solution methods does not depend only on the size of the matrix representing the window but also on its mathematical properties.

The problems mentioned above can be circumvented by calculating *τ*_1_, ..., *τ*_*r *_during the construction of the window *W*_*j *_as follows. We compute the number of the states that are significant at time *t*_*j*-1 _and pass it to *ContDetApprox *in line 9 (see Table 1). We run the while loop in Algorithm *ContDetApprox *(see Table 1, left) until (1) the window has at least a certain size and (2) the number of states in the window exceeds twice the number of the states that are significant at time *t*_*j*-1_. The first condition ensures that the window exceeds a certain minimum size of, say, 500 states. The second condition ensures that the new window is just big enough to move the probability mass to a region outside of *S*_*j*_. More precisely, it ensures that the sets *S*_1_, *S*_2_,...are not overlapping and that subsequent sets are located next to each other (as illustrated in Figure 1). Note that this ensures that the resulting window does not contain many states that are only significant during a much shorter time interval.

On termination of the while-loop, we pass the value of the variable *time *from *ContDetApprox *to *sWindow *and set *τ*_*j *_to the value of *time*. Obviously, in *sWindow *we add a variable representing the time elapsed so far, and the for loop in line 2 is replaced by a while loop that stops when the time elapsed so far exceeds *t*. Later, we present experimental results of the sliding window method where we use adaptive time steps in the way described above.

### Numerical solution methods

In this section, we present the theoretical basis of two numerical solution algorithms, namely the uniformization method and the Krylov subspace method. We approximate a global solution of the CME (cf. Eq. (5)), as well as the local solutions that are required in line 13 of Algorithm *sWindow *(see also Eq. (7)).

#### Uniformization

The uniformization method goes back to Jensen [34] and is also referred to as Jensen's method, randomization, or discrete-time conversion. In the performance analysis of computer systems, this method is popular and often preferred over other methods, such as Krylov subspace methods and numerical integration methods [19,39]. Recently, uniformization has also been used for the solution of the CME [40-42].

##### Global uniformization

Let (*X*(*t*), *t *∈ ℝ_≥0_) be a CTMC with finite state space *S*. The basic idea of uniformization is to define a discrete-time Markov chain (DTMC) and a Poisson process. The DTMC is stochastically identical to *X*, meaning that it has the same transient probability distribution if the number of steps within [0, *t*) is given, and the Poisson process keeps track of the time as explained below.

Recall that *λ*_*x *_is the exit rate of state *x *∈ *S*, and *I *is the identity matrix. We define a *uniformization rate λ *such that *λ *≥ max_*x*∈*S *_*λ*_*x *_and construct , the transition matrix of the DTMC associated with *X*. Note that a diagonal entry in *P *defines the self-loop probability 1 - *λ*_*x*_/*λ *of a state *x*, which is nonzero if and only if *λ *> *λ*_*x*_. For *k *≥ 1, the stochastic matrix *P*^*k *^contains the *k*-step transition probabilities and, if ***p***^(0) ^is the initial distribution of *X*, the vector ***w***^(*k*) ^= ***p***^(0)^*P*^*k *^contains the state probabilities after *k *steps in the DTMC. The number of steps within time interval [0, *t*) has a Poisson distribution with parameter *λt*, i.e.,(12)

Now, the solution of the transient state probabilities in Eq. (5) can be rewritten as [19,32,43](13)

Eq. (13) has nice properties compared to Eq. (5). There are no negative summands involved, as *P *is a stochastic matrix and *λ *> 0. Moreover, ***w***^(*k*) ^can be computed inductively by(14)

If *P *is sparse, ***w***^(*k*) ^can be calculated efficiently even if the size of the state space is large. Lower and upper summation bounds *L *and *U *can be obtained such that for each state *x *the truncation error [44](15)

can be a priori bounded by a predefined error tolerance ϵ > 0. Thus, ***p***^(*t*) ^can be approximated with arbitrary accuracy by(16)

as long as the required number of summands is not extremely large.

##### Time complexity and stiffness

As *λt *grows the Poisson distribution flattens, and the left truncation point *L *in Eq. (16) grows linearly in *λt*, while the number of significant Poisson probability terms is [44]*O*(). If the vectors ***w***^(*L*)^, ***w***^(*L*+1)^, ..., ***w***^(*U*) ^are computed using *U *matrix-vector multiplications (cf. Eq. (14)), then the complexity of the uniformization procedure is *O*(*νλt*) where *ν *is the number of nonzero elements in *P*.

All analysis methods (simulation-based or not) encounter serious difficulties if the underlying model is *stiff*. In a stiff model the components of the underlying system act on time scales that differ by several orders of magnitude and this arises in various application domains, especially in systems biology. For a stiff model, the uniformization rate *λ *≥ max_*x*∈*S *_*λ*_*x *_will correspond to the fastest time scale. By contrast, a significant change of the slow components can be observed only during a period of time that corresponds to the slowest time scale. The uniformization method is then extremely time consuming because of a very large *stiffness index *[45]*t*·max_*x*∈*S *_*λ*_*x*_.

In the sequel, we show how uniformization can be applied in a local fashion such that stiffness has a less negative effect on the performance of the method. In other words, the sliding window technique enables uniformization to perform well even for stiff systems.

##### Local uniformization

We now combine uniformization and the sliding window method. Assume that *S *may be infinite, and that we iteratively apply uniformization to solve Eq. (7). More specifically, in line 13 of Algorithm *sWindow *(see Table 1, right), we invoke the uniformization method to approximate

Thus, *P*_*j *_= *I *+  is a substochastic transition matrix, where *λ*_*j *_= . By using the same calculation as in Eq. (16), we obtain a substochastic vector(17)

where *L *and *U *are the truncation points depending on *λ*_*j*_*τ*_*j*_, and . Moreover, as *λ*_*j *_depends only on *W*_*j*_, the uniformization rate is usually smaller than the global one, sup_*x*∈*S *_*λ*_*x*_, which means that fewer terms are required in Eq. (17) than in Eq. (16).

The computational complexity of the whole procedure is *O*(), and thus, we save computation time, compared to global uniformization, if , where *λ *= sup_*x*∈*S *_*λ*_*x *_and *ν*_*j *_is the number of nonzero elements in *P*_*j*_.

#### Krylov subspace

Krylov subspace methods are widely used for large eigenvalue problems, for solving linear equation systems, and also for approximating the product of a matrix exponential and a vector [46,47]. We are interested in the latter approximation and show how it can be used to solve the CME, either in a global fashion or in combination with the sliding window method. Recently, Krylov subspace methods have been applied to the CME by Sidje et al. [21].

##### Global Krylov subspace method

Recall that a global solution of the CME is given by ***p***^(*t*) ^= ***p***^(0)^*e*^*Qt*^. In the sequel, we describe the approximation of *e*^*tA*^***v***, where *A *is an *N *× *N *square matrix and ***v ***is a column vector of length *N*. We obtain an approximation of ***p***^(*t*) ^by choosing *A *= (*Q*)^*T *^and ***v ***= (***p***^(0)^)^*T*^. Let us first assume that *t *= 1. The main idea is to generate a basis of the *m*-th Krylov subspace

and to seek an approximate solution for *e*^*A*^***v ***from this subspace. Let *q*^min ^be the nonzero monic polynomial of lowest degree such that *q*^min^(*A*)***v ***= 0. We choose *m *∈ ℕ such that the degree of *q*^min ^is greater or equal to *m*. In this case, the vectors ***v***, *A****v***, ..., *A*^*m*-1^***v ***are linearly independent, and for every element ***x ***∈ *K*_*m *_there exists a polynomial *q *of degree at most *m *- 1 with ***x ***= *q*(*A*)***v***. Note that in practice we choose *m *= 30 or *m *= 20, because the degree of *q*^min ^is usually greater than 30. However, if not, the problem can be solved *exactly *in the *d*-th Krylov subspace, where *d *is the degree of *q*^min^. Working directly with the basis {***v***, *A****v***, ..., *A*^*m*-1^***v***} is numerically unstable. Therefore, we construct an orthonormal basis {***v***_1_, ***v***_2_, ..., ***v***_*m*_} for *K*_*m *_by applying Arnoldi's algorithm to ***v***, *A****v***, ..., *A*^*m*-1^***v***. Let *H*_*m *_be the *m *× *m *upper Hessenberg matrix computed by the Arnoldi algorithm and let *h*_*m*+1, *m *_be the last normalization value. By *V*_*m *_we denote the *N *× *m *matrix with column vectors ***v***_1_, ***v***_2_, ..., ***v***_*m*_. Then(18)

where ***e***_*k *_is a column vector of appropriate size whose *k*-th component is one and all other components are zero. Intuitively, Eq. (18)(b) states that *H*_*m *_is the matrix projection of *A *onto *K*_*m *_w.r.t. the basis defined by *V*_*m*_. An approximation of *e*^*A*^***v ***in *K*_*m *_expressed using *V*_*m *_is *e*^*A*^***v ***≈ *V*_*m*_***y***, where ***y ***is a vector of size *m*.

We choose(19)

which yields the approximation error [46](20)

where *ρ *= ||*A*||_2 _is the spectral norm of *A*. The approximation in Eq. (19) still involves the computation of the matrix exponential of *H*_*m*_, but as *H*_*m *_is of small dimension and has a particular structure (upper Hessenberg), this requires a smaller computational effort. For the matrix exponential of small matrices, methods such as Schur decomposition and Padé approximants may be applied [31].

Assume now that the time instant *t *is arbitrary, i.e., we want to approximate *e*^*tA*^***v ***for some *t *> 0. In order to control the approximation error, we calculate *e*^*tA*^***v ***stepwise by exploiting that  for *τ*_1_, *τ *_2 _≥ 0. For a step size *τ*, we approximate *e*^*τA*^***v ***by ||***v***||_2 _ because the Krylov subspaces associated with *A *and *τA *are identical and . It follows from Eq. (20) that we have a small error bound if ||*Aτ*||_2 _is small.

To summarize, the Krylov subspace method approximates *e*^*At*^***v ***by an iteration stepping forward in time with dynamically chosen step sizes *τ*_1_, *τ*_2_,.... In each iteration step, we compute a vector

where initially ***u***_0 _= ***v***. The matrices  and  result from the *i*-th execution of Arnoldi's algorithm for the construction of an orthonormal basis of the subspace *Span*{***u***_*i*-1_, *A****u***_*i*-1_, ..., *A*^*m*-1^***u***_*i*-1_}. When the elapsed time equals *t*, we obtain an approximation of *e*^*At*^***v***.

For the step size of the Krylov subspace method, a popular heuristic is to choose *τ*_*i*+1 _depending on an estimate of the error ϵ_*i *_of the previous step. Let *tol *> 0 be an a priori specified tolerance. A common scheme consists of three steps [36]. (1) Define , (2) compute ***u***_*i *_and the error estimation ϵ_*i*_. (3) If ϵ_*i *_> 1.2 *tol *reject ***u***_*i*_, replace ϵ_*i*-1 _with ϵ_*i*_, and go to step (1). For our experimental results, we used the Expokit software [48] where the small exponential, , is computed via the irreducible Padé approximation [49].

##### Local Krylov subspace method

Assume now that we use the Krylov subspace method in line 13 of Algorithm *sWindow *(see Table 1, right), to approximate  (cf. Eq. (7)). By letting ***v ***= , *A *= , and *t *= *τ*_*j *_we can apply the same procedure as in the global case. Note that this yields a nested iteration because the time steps *τ*_*j *_are usually much bigger than the time steps of the Krylov subspace method. For the Krylov subspace method, using the matrix *Q*_*j *_instead of *Q *offers important advantages. The Arnoldi process is faster as *Q*_*j *_usually contains fewer nonzero entries than *Q*. As well, the sliding window method is likely to provide matrices with a smaller spectral norm ||*Q*_*j*_||_2_. This allows for bigger time steps during the Krylov approximation, as can be seen in our experimental results.

### Experimental results

**Figure 2:**
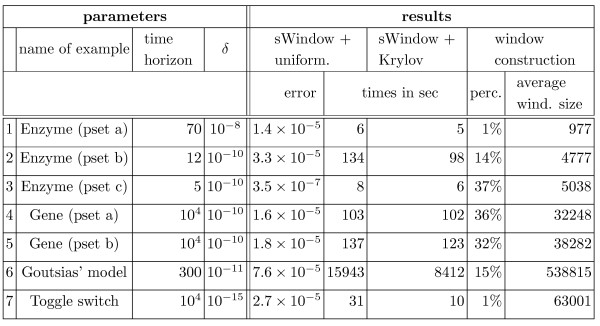
**Parameters and results of the sliding window method**.

We coded both algorithms in Table 1 in C++ and ran experiments on a 3.16 GHz Intel dual-core Linux PC. We discuss experimental results that we obtained for Example 1 and Example 2, as well as Goutsias' model [50] and a bistable toggle switch [51]. Goutsias' model describes the transcription regulation of a repressor protein in bacteriophage *λ *and involves six different species and ten reactions. The bistable toggle switch is a prototype of a genetic switch with two competing repressor proteins and four reactions. All results are listed in Figure 2.

As explained in detail below, we also implemented the method proposed by Burrage et al. [21] in order to compare it to our algorithm in terms of running time and accuracy. Moreover, for finite examples we compare our method to a global analysis, i.e. where in each step the entire state space is considered. We do not compare our method to Gillespie simulation or approximation methods based on the Fokker-Planck equation. The former method provides only estimates of the probability distribution and becomes infeasible if small probabilities are estimated [52]. The latter type of methods do not take into account the discreteness of the molecule numbers and are known to provide bad approximations in the case of small populations as considered here [53].

#### Parameters

We fixed the input ϵ = 10^-8 ^of Algorithm *sWindow *for all experiments (see Table 1, right). We chose the input *δ *in a dynamical fashion to ensure that in the *j*-th step we do not lose more probability than 10^-5^·*τ*_*j*_/(*t*_*r *_- *t*_0_) by restricting to significant states, that is, we decrease *δ *until after line 4 of Algorithm *sWindow *the set *S*_*j *_contains at most 10^-5^· less probability than the former set *S*_*j*-1_. In Figure 2, we list the average value that we used for *δ*.

In the sequel, we give details about the parameters used for the results that we obtained for Example 1 and Example 2. For the remaining two examples, we list the corresponding chemical reactions and the parameters that we chose for the results in Figure 2.

##### Enzyme example

We tried different parameter sets, referred to as pset a)-c), for Example 1 (see Figure 2). For parameter combination a) we have *c*_1 _= *c*_2 _= 1, *c*_3 _= 0.1 and start with 1000 enzymes and 100 substrates. In this case the number of reachable states is 5151. For parameter set b) and c) we have *c*_1 _= *c*_2 _= *c*_3 _= 1 and start with 100 enzymes and 1000 substrates and 500 enzymes and 500 substrates, which yields 96051 and 125751 reachable states, respectively. Each time we choose the time horizon according to the time until most of the probability mass is concentrated in the state in which all substrate molecules are transformed into products. For the time steps *τ*_*j *_in Algorithm *sWindow*, we apply the condition described above.

We consider four branches for the iteration in Eq. (11) in order to determine upper and lower bounds on the state variables. (1) To obtain an estimate for the maximal number of complex molecules (and a minimum for the enzyme population), we enforce more reactions of type *R*_1 _than on average (*κ*_1 _= ), and fewer of types *R*_2 _and *R*_3 _(*κ*_3 _=  and *κ*_2 _= ). (2) By considering fewer reactions of type *R*_1 _(*κ*_1 _= ), and more of types *R*_2 _and *R*_3 _(*κ*_3 _=  and *κ*_2 _= ) the complex population becomes minimal (and the enzyme population maximal). (3) An estimate for the minimal number of type *P *molecules (and the maximal number of type *S *molecules) is obtained by enforcing more reactions of type *R*_2 _(*κ*_2 _= ), and fewer of types *R*_1 _and *R*_3 _(*κ*_1 _=  and *κ*_3 _= ). (4) Finally, more reactions of types *R*_1 _and *R*_3 _(*κ*_1 _=  and *κ*_3 _= ), and fewer of type *R*_2 _(*κ*_2 _= ) gives a maximal increase of the number of product molecules (and minimizes the number of substrate molecules).

**Figure 3:**
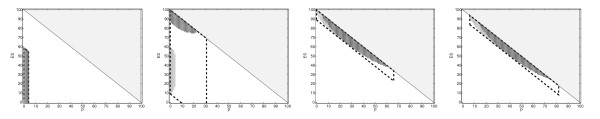
**Parallelogram shape**. For the enzyme reaction example, the set of reachable states is finite and delimited by the diagonal, which is represented by the line *ES *= 100 - *P *if 100 is the initial number of enzyme molecules. For certain parameter sets, the window has a parallelogram shape which corresponds to the direction in which the probability mass is moving.

For the enzyme example, if the initial conditions are fixed a state is uniquely determined by at least two entries, say, the population of complex and product molecules. However, a rectangular window shape yields poor results if the expected number of complex molecules is high. The reason is that in this case the probability mass is located on a diagonal (cf. Figure 3). If the set of significant states is captured by a rectangular window it may contain many states that are not significant. This problem can be circumvented by considering bounds for all state variables during the window construction as well as the conservation laws. More precisely, the parallelogram in Figure 3 is constructed by computing for each value  of *P *upper and lower bounds on *ES *by  and , where *y *= (*y*_1_, *y*_2_, 0, 0) is the initial population vector and  and  are the upper and lower bounds on the populations of *E*, *S*, *ES*, and *P*.

Note that the parallelogram in Figure 3 was induced by the conservation laws of the system. In general, conservation laws should be taken into account since otherwise the window may be inconsistent with the conservation laws, i.e. it may contain states that are not reachable.

##### Gene expression example

In Figure 2 we present results for Example 2. The difference between parameter set a) and parameter set b), referred to as pset a) and pset b), is that for a) we start with the empty system and for b) we start with 100 mRNA molecules and 1000 proteins. For both variants, we choose rate constants *c*_1 _= 0.5, *c*_2 _= 0.0058, *c*_3 _= 0.0029, *c*_4 _= 0.0001. The time steps that we use are determined by the condition in the section on time intervals. Note that we cannot solve this example using a global method because the number of reachable states is infinite. The column *error *contains the total error *η*_*r *_(see Eq. (8)) and *times in sec *refers to the running time in seconds. In column *perc*. we list the percentage of the total running time that was spent for the window construction. The column *average wind*. *size *refers to the average number of states in the window.

For the gene expression example, we use four branches: We maximize the number of mRNA molecules by choosing  and  and minimize it with  and . Reactions *R*_2 _and *R*_4 _are irrelevant for this species. We maximize the protein population by choosing , and  and minimize it with , and .

##### Goutsias' model

The model, referred to as Goutsias' model in Figure 2, is composed by the following chemical reactions [50]:

1:   RNA   →   RNA + M

2:   M   →   ∅

3:   DNA.D   →   RNA + DNA.D

4:   RNA   →   ∅

5:   DNA + D   →    DNA.D

6:   DNA.D   →   DNA + D

7:   DNA.D + D   →   DNA.2D

8:   DNA.2D   →   DNA.D + D

9:   M + M   →   D

10:   D   →   M + M

We used the same kinetic constants as Goutsias [50] and Sidje et al. [21], as well as the same initial state.

**Figure 4:**
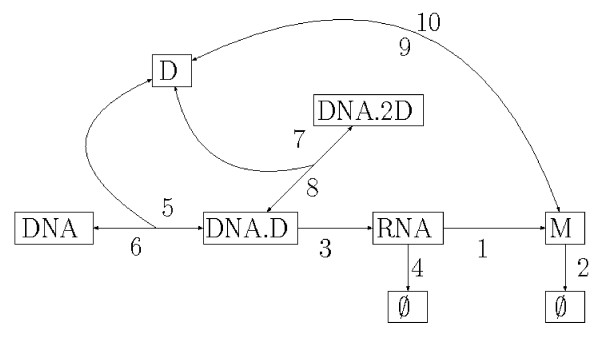
**Dependencies between the reactions of Goutsias' model**.

**Figure 5:**
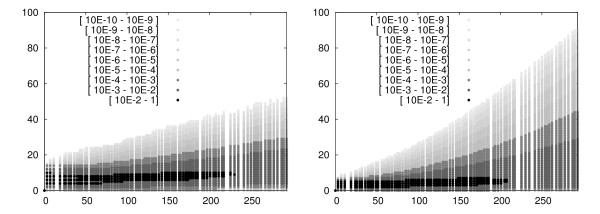
**The probability distribution of monomers (left) and dimers (right) during the time interval [0, 300)**.

Below, we list the branches for upper bounds on the state variables. Lower bounds are obtained if the opposite combination is considered, respectively. We refer to Figure 4 for an illustration of the dependencies between the reactions that simplifies the choice of the branches. We maximize the RNA population by choosing the combination . We maximize the monomer population by choosing the combination . We maximize the number of dimer molecules by choosing the combination . Note that although dimers are consumed by reaction 5, choosing  maximizes the number of dimers in the system. This is because reaction 5 is necessary to produce monomers and therefore also dimers. We never run out of memory with the sliding window method, but the running times can be huge for a long time horizon. The reason is that the windows are large since the system contains many monomers and dimers at later time instances. For the results in Figure 2 we considered the system till time *t *= 300, whereas for Sidje et al. [21], the longest time horizon is *t *= 100. In Figure 5 we plot the distribution of the species *M *and *D*.

##### Bistable toggle switch

The toggle switch involves two chemical species *A *and *B *and four reactions. Let *x *= (*x*_1_, *x*_2_) ∈ . The reactions are ∅ → *A*, *A *→ ∅, ∅ → *B*, *B *→ ∅ and their propensity functions *α*_1_, ..., *α *_4 _are given by *α*_1_(*x*) = *c*_1_/(*c*_2 _+ ), *α*_2_(*x*) = *c*_3_·*x*_1_, *α*_3_(*x*) = *c*_4_/(*c*_5 _+ ), *α*_4_(*x*) = *c*_6_·*x*_2_. Note that in this example the propensity functions are not of the form described in Eq. 1. For our experimental results, we chose the same parameters as Sjöberg et al. [23], that is, *c*_1 _= *c*_4 _= 3·10^3^, *c*_2 _= *c*_5 _= 1.1·10^4^, *c*_3 _= *c*_6 _= 0.001, and *β *= *γ *= 2. The initial distribution is a Gaussian distribution (*μ*, *σ*^2^) with *μ *= (133, 133)^*T *^and . We consider the obvious four branches each of which is intended to minimize/maximize one of the two components. The branch minimizing *A *for example will have less of the first reaction and more of the second.

## Discussion

In this section, we discuss our algorithm w.r.t. accuracy and running time where we consider different solution methods and different time step mechanisms. Moreover, we compare our method with a global analysis.

### Accuracy

The column labeled by *error *in Figure 2 shows the total error *η*_*r *_(cf. Eq. (8)) of the sliding window method plus the uniformization error (which is bounded by ϵ = 10^-8^). The error using the Krylov subspace method instead yields the same accuracy because for both, uniformization and the Krylov subspace method, the error bound is specified a priori. For all examples, the total error does not exceed 1 × 10^-4^, which means that not more than 0.01 percent of the probability mass is lost during the whole procedure. It would, of course, be possible to add an accuracy check in the while loop of Algorithm *sWindow*, expand the current window if necessary, and recalculate. But as the method consistently returns a small error, this has been omitted.

We also considered relative errors, that is,  for states *x *∈ *W*_*j *_with  > 10^-5^. We approximate the value  by solving Eq. (13) via global uniformization, where we use truncation error ϵ = 10^-8^. Since this is only possible if the state space is finite, we compared relative errors only for the enzyme example. Our calculations show that the relative errors are always smaller than 10^-4^.

In order to support our considerations in the window construction section, we carried out experiments in which we exclusively chose the average in line 17 of Algorithm *ContDetApprox *(see Table 1, left). More precisely, for the construction of the window we do not consider the deviations in the numbers of reactions but only the average number. In this case, we called the method *ContDetApprox *with input 2*τ *to make sure that on average the probability mass moves to the center of the window and not too close to the borders. For this configuration, the total error is several orders of magnitude higher, e.g., for parameter set a) of the enzyme example the total error is 0.0224.

Finally, we test the size of the windows constructed in lines 7-10 of Algorithm *sWindow*. We change Algorithm *sWindow *by decreasing the size of the window by 5% between lines 10 and 11. In this case, the total error *η*_*r *_increases. For instance, *η*_*r *_= 0.35% for parameter set a) of the enzyme example. These results substantiate that the size and the position of the sliding window is such that the approximation error is small whereas significantly smaller windows result in significantly higher approximation errors.

### Running time

For the time complexity analysis, we concentrate on three main issues.

• Sliding window method vs. global analysis: We compare the sliding window method with a global solution in one step, and with another window method, where the size of the window is doubled if necessary.

• Solution method (uniformization vs. Krylov subspace method): In Algorithm *sWindow*, we vary the solution method by exchanging uniformization with the Krylov subspace method.

• Time intervals (equidistant vs. adaptive time steps): We use different methods to determine the length *τ*_*j *_of the next time step in line 3 of Algorithm *sWindow*.

#### Sliding window method vs. global analysis

**Figure 6:**
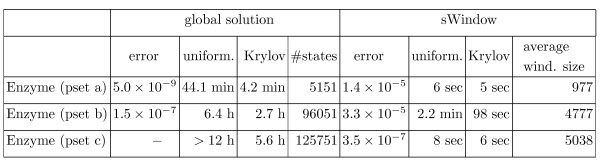
**Sliding window method vs. global analysis for the finite enzyme example**.

We used the enzyme example to compare the sliding window solution with a global solution (global uniformization and global Krylov subspace method), since it has a finite state space. Note that all other examples cannot solved using a global method since their state space is infinite. We list the time needed for the computation of  (cf. Eq. (3)) with the global method in Figure 6.

Observe that the total error of the global uniformization method is smaller (compare the columns labeled by *error*) since the only error source is the truncation of the infinite sum in Eq. (13). In the column with heading *#states *we list the number of states that are reachable. During the global solution we consider all reachable states at all time whereas in the sliding window method the average number of states considered during a time step is much smaller. This is the main reason why the sliding window method is much faster. Moreover, in the case of uniformization, the rate for global uniformization is the maximum of all exit rates, whereas for local uniformization, we take the maximum over all states in the current window. Note that the global maximum can be huge compared to the local maxima. This explains the bad performance of the global uniformization method. When the Krylov subspace method is used for a global solution, the running times of the global solutions are also higher than the times of the local Krylov subspace method (sliding window method combined with the Krylov subspace method). Again, the reason is that a smaller number of states is considered during the sliding window iteration. Moreover, the matrices *Q*_*j *_have numerical properties that facilitate the use of bigger, and thus, fewer time steps. The total number of iteration steps used to solve Eq. (6) with the Krylov subspace method and the sliding window method is indeed small when compared to the global Krylov subspace method (on average around 20 times fewer steps).

We now focus on a comparison between our sliding window method and another local method, called *doubling window method*. For the latter, we compute the probability vectors in a similar way as Sidje et al. [21]. We start with an initial window and apply the Krylov algorithm. We do not iterate over the time intervals [*t*_*j*-1_, *t*_*j*_) but use the step sizes of the Krylov subspace method. After each time step, we remove those parts of the window that will not be used for the remaining calculations. We expand the size of the window if the error exceeds a certain threshold. Since the performance of the method depends heavily on the initial window and the directions in which a window is expanded, we start initially with the same window as the sliding window method and expand always in the directions that are most advantageous for the computation. For this we used information about the direction in which the probability mass is moving that we obtained from experiments with the sliding window method. The expansion of a window is realized by doubling the length of all of its edges.

We applied the doubling window method to the enzyme example and the gene expression. For all parameter sets that we tried, the sliding window method outperforms the doubling window method w.r.t. running time (with an average speed-up factor of 5). The total number of iterations of the Krylov subspace approximation is up to 13 times smaller in the case of the sliding window method compared to the doubling window method (with an average of 6.5). Note that for arbitrary systems the doubling window method cannot be applied without additional knowledge about the system, i.e., it is in general not clear, in which direction the window has to be expanded.

Our results indicate that the sliding window method achieves a significant speed-up compared to global analysis, but also compared to the doubling window method. Moreover, while global analysis is limited to finite-state systems and the doubling window methods requires additional knowledge about the system, our method can be applied to any system where the significant part of the probability mass is located at a tractable subset of states. If the dimension of the system is high, then the significant part of the probability mass may be located at intractably many states and in this case the memory requirements of our algorithm may exceed the available capacity.

### Solution method

During the sliding window iteration different solution methods can be applied in line 13 of Algorithm *sWindow*. We concentrate on the uniformization method and on the Krylov subspace method. The running times in Figure 2 (compare the columns labeled by *sWindow + uniformization *with the columns labeled by *sWindow + Krylov*) show that the Krylov subspace method performs better (average speed-up factor of around 1.5). The reason is that the Krylov subspace method is more robust to stiffness than uniformization. For non-stiff systems, uniformization is known to outperform the Krylov subspace method [19,39]. However, since biochemical network models are typically stiff, the Krylov subspace method seems to be particularly well suited in this area.

### Time intervals

In order to confirm our considerations in the section on time intervals, we also applied the sliding window method using equidistant time steps. For all examples, using equidistant time steps results in longer computation times compared to using adaptive time steps (with an average speed-up factor of 3.5). A adaptive choice of the time steps has also the advantage that we can control the size of the windows and avoid that the memory requirements of the algorithm exceed the available capacity.

## Conclusions

The sliding window method is a novel approach to address the performance problems of numerical algorithms for the solution of the chemical master equation. It replaces a global analysis of the system by a sequence of local analyzes. The method applies to a variety of chemically reacting systems, including systems for which no upper bound on the population sizes of the chemical species is known a priori. The proposed method is compatible with all existing numerical algorithms for solving the CME, and also a combination with other techniques, such as time scale separation [26,27], is possible.

We demonstrated the effectiveness of our method with a number of experiments. The results are promising as even systems with more than two million states with significant probability can be solved in acceptable time. Moreover, for examples that are more complex than those presented here, it is often sufficient to consider only a relatively small part of the state space. The number of molecules in the cell is always finite and, usually, a biochemical system follows only a small number of different trends. Stated differently, it is rarely the case that in biochemical systems a large number of different scenarios have significant likelihoods. Thus, we expect that the sliding window method can be successfully applied to systems with many chemical species and reactions as long as the significant part of the probability mass is always located at a tractable subset of states. In addition, further enhancements are possible, such as a splitting of the windows, which will be particularly useful for multi-stable systems. Moreover, we plan to automate our algorithm in a way that besides the initial conditions and the set of reactions no further input from the user is necessary, such as combinations of reactions that maximize/minimize certain populations.

## Authors' contributions

VW and TAH designed the research. VW, RG, MM, and TAH developed the algorithm and the implementation was carried out by RG and MM. VW, MM, and TAH wrote the manuscript, which has been read and approved by all authors.
